# Combined Analysis of *Interleukin-10* Gene Polymorphisms and Protein Expression in Children With Cerebral Palsy

**DOI:** 10.3389/fneur.2018.00182

**Published:** 2018-03-22

**Authors:** Lei Xia, Mingjie Chen, Dan Bi, Juan Song, Xiaoli Zhang, Yangong Wang, Dengna Zhu, Qing Shang, Falin Xu, Xiaoyang Wang, Qinghe Xing, Changlian Zhu

**Affiliations:** ^1^Henan Key Laboratory of Child Brain Injury, Third Affiliated Hospital of Zhengzhou University, Zhengzhou, China; ^2^Institute of Biomedical Science, Children’s Hospital, Fudan University, Shanghai, China; ^3^Department of Pediatrics, Zhengzhou Children’s Hospital, Zhengzhou, China; ^4^Perinatal Center, Institute of Neuroscience and Physiology, University of Gothenburg, Gothenburg, Sweden; ^5^Shanghai Center for Women and Children’s Health, Shanghai, China; ^6^Center for Brain Repair and Rehabilitation, Institute of Neuroscience and Physiology, University of Gothenburg, Gothenburg, Sweden

**Keywords:** cerebral palsy, cytokine, inflammation, interleukin-10, single nucleotide polymorphisms

## Abstract

**Background:**

Interleukin-10 (IL-10) is an important anti-inflammatory and immunosuppressive cytokine, and it has indispensable functions in both the onset and development of inflammatory disorders. The association between persistent inflammation and the development of cerebral palsy (CP) has attracted much attention.

**Objective:**

The purpose of this study was to investigate whether *IL-10* gene polymorphisms and plasma protein expression are associated with CP and to analyze the role of IL-10 in CP.

**Methods:**

A total of 282 CP patients and 197 healthy controls were genotyped for *IL-10* polymorphisms (rs1554286, rs1518111, rs3024490, rs1800871, and rs1800896). Among them, 95 CP patients and 93 healthy controls were selected for plasma IL-10 measurement.

**Results:**

The differences in the rs3024490 (*p* = 0.033) and rs1800871 (*p* = 0.033) allele frequencies of *IL-10* were determined between CP patients and controls. The frequencies of allele and genotype between CP patients with spastic tetraplegia and normal controls of *IL-10* polymorphisms showed significant differences for rs1554286, rs151811, rs3024490, rs1800871, and rs1800896 (*p_allele_* = 0.015, 0.009, 0.006, 0.003, and 0.006, *p_genotype_* = 0.039, 0.018, 0.027, 0.012, and 0.03, respectively). The plasma IL-10 protein level in CP patients was higher than normal controls (9.13 ± 0.77 vs. 6.73 ± 0.63 pg/ml, *p* = 0.017). IL-10 polymorphisms and protein association analysis showed that the TT genotype had higher plasma IL-10 protein levels compared to the GG + GT genotype at rs3024490 (11.14 ± 7.27 vs. 7.44 ± 6.95 pg/ml, *p* = 0.045, respectively) in CP cases.

**Conclusion:**

These findings provide an important contribution toward explaining the pleiotropic role of IL-10 in the complex etiology of CP.

## Introduction

Cerebral palsy (CP) comprises a group of disorders affecting movement and posture due to non-progressive lesions or abnormalities in the immature brain, and these can lead to costly and life-long disability (1, 2). Studies have identified a number of risk factors for the development of CP. During the last decade, intrauterine infection and inflammation have been recognized as the most common causes of preterm delivery and white-matter brain injury such as periventricular leukomalacia (PVL) and subsequent development of CP (3–6). It is also known that the activation of inflammation plays a key role in the mechanisms of intrauterine infection-triggered brain damage (7, 8).

It is well known that the pro-inflammatory cytokine level in the amniotic fluid or in neonatal blood is directly related to the risk of developing CP (3, 9), and high levels of expression of TNFα, IL-1β, and IL-6 increase the risk for development of brain lesions with PVL (7, 10). IL-17 has also been reported to act synergistically with TNF and IL-1 and to play a notable role in inducing and mediating pro-inflammatory responses (11–13). These pro-inflammatory cytokines are responsible for initiating inflammation in response to tissue damage, while anti-inflammatory cytokines are released to limit the sustained or excessive inflammatory response (14). IL-10 is a main anti-inflammatory cytokine and has been suggested to play a crucial role in neuronal homeostasis and cell survival (15, 16). IL-10 plays a protective role in microglial cultures after a pro-inflammatory insult (17) and in rat pups born to dams infected with *Escherichia coli* (18). However, several studies have questioned the perception of IL-10 solely as an immunosuppressive cytokine because IL-10 can also stimulate immune responses by promoting the proliferation and cytotoxic activity of natural killer cells and CD8+ T-cells (19) as well as the survival, proliferation, differentiation, MHC class II expression, and antibody production of B-cells (20, 21). Increasing evidence indicates that IL-10 is involved in both the onset and development of inflammatory diseases. In a study of very low birth weight children suspected of having late-onset sepsis, the expression of the anti-inflammatory cytokines IL-4 and IL-10 was significantly elevated (22). Together, these studies indicate that the biological activity of IL-10 is variable and results in protective or pathogenic effects at different stages of disease and that IL-10 might play an important role in the pathogenesis of CP.

The protein expression of cytokines is regulated by genetic variants in the cytokine genes (15, 23), and accumulating evidence suggests that inherited cytokine single nucleotide polymorphisms (SNPs) contribute to increased risk of CP (24, 25). In following with this, it has been reported that SNPs within the coding and promoter regions of the *IL-10* gene can affect the expression and secretion of this cytokine (26, 27). Considering the potential role of *IL-10* in the etiology of CP, the present research sought to evaluate the possible association of *IL-10* SNPs and IL-10 plasma levels with susceptibility to CP in a Chinese population.

## Materials and Methods

### Subjects

A total of 282 CP patients and 197 healthy controls were enrolled (Table 1). We collected all CP patients between 1 July 2010 and 31 May 2012 from the 3rd Affiliated Hospital of Zhengzhou University and Zhengzhou Children’s Hospital, which included 98 girls (34.8%) and 184 boys (65.2%) with a mean age ± SD of 16.2 ± 12.7 months. The diagnosis of CP patients was made by child neurologists according to the guidelines proposed by the Surveillance of CP in Europe network (28) through clinical examination or medical records, including brain imaging. The healthy controls [42 girls (21.3%) and 155 boys (78.7%) with a mean age ± SD of 24.0 ± 16.4 months] were enrolled during their physical examination from the same hospitals. Of these, 95 CP patients (56 boys and 39 girls) and 93 healthy controls (78 boys and 15 girls) were selected for the assay of IL-10 protein level (Table 2), and the mean age ± SD was 20.8 ± 14.4 and 21.6 ± 13.8 months, respectively. All subjects were Han Chinese as reported by their parents. All of the subjects, including both CP patients and controls, with myopathy, metabolic anomalies, or infections were excluded because of the genetic and familial factors that are associated with CP. Approval for the study was obtained from the ethics committee of Zhengzhou University in accordance with the Helsinki Declaration (201002006). Written informed consent was obtained from at least one of the parents after fully explaining the procedure.

**Table 1:** Sample description for gene polymorphism analysis.

Characteristic	CP cases		Control	
		
	Total (%)	M/F (*n*)	Total (%)	M/F (*n*)
**Type of CP**				
CP with Spastic Tetraplegia	123 (43.6)	76/47	–	–
CP with Spastic Deplegia	72 (25.5)	47/25	–	–
CP with Spastic Hemiplegia	27 (9.6)	24/3	–	–
CP without Spastic	60 (21.3)	37/23	–	–

Total	282 (100)	184/98	197 (100)	155/42

**Birth asphyxia**				
No asphyxia	183 (64.9)	113/70	187 (94.9)	148/39
Asphyxia	99 (35.1)	71/28	10 (5.1)	7/3

Total	282 (100)	184/98	197 (100)	155/42

**Complication**				
With PVL	41 (14.5)	28/13	–	–
Without PVL	241 (85.5)	156/85	–	–
With MR	177 (62.8)	114/63	–	–
Without MR	105 (37.2)	70/35	–	–
With HIE	93 (33.0)	66/27	–	–
Without HIE	189 (67.0)	118/71	–	–
**Maternal factors**				
PROM	52 (18.4)	35/17	14 (7.1)	10/4
No PROM	230 (81.6)	149/81	183 (92.9)	145/38
TPL	49 (17.4)	33/16	23 (11.7)	18/5
No TPL	233 (82.6)	151/82	174 (88.3)	137/37
PIH	19 (6.7)	14/5	13 (6.6)	12/1
No PIH	263 (93.3)	170/93	184 (93.4)	143/41

*CP, cerebral palsy; M, male; F, female; HIE, hypoxia ischemia encephalopathy; PVL, periventricular leukomalacia; MR, mental retardation; PROM, premature rupture of membrane; TPL, threatened premature labor; PIH, pregnancy-induced hypertension*.

**Table 2:** Sample description for cytokine production analysis.

Characteristic	CP cases		Control	
		
	Total (%)	M/F (*n*)	Total (%)	M/F (*n*)
**Type of CP**				
CP with spastic tetraplegia	23 (24.2)	11/12	–	–
CP with spastic deplegia	35 (36.8)	21/14	–	–
CP with spastic hemiplegia	8 (8.4)	7/1		
CP without spastic	29 (30.5)	17/12		

Total	95 (100)	56/39	93 (100)	78/15

**Birth asphyxia**				
No asphyxia	58 (61.1)	31/27	91 (97.8)	77/14
Asphyxia	37 (38.9)	25/12	2 (2.2)	1/1

Total	95 (100)	56/39	93 (100)	78/15

**Complication**				
With PVL	23 (24.2)	14/9	–	–
Without PVL	72 (75.8)	42/30	–	–
With MR	75 (78.9)	40/35	–	–
Without MR	20 (21.1)	16/4	–	–
With HIE	7 (7.4)	5/2	–	–
Without HIE	88 (92.6)	51/37	–	–
**Maternal factors**				
PROM	20 (21.1)	11/9	7 (7.5)	6/1
No PROM	75 (78.9)	47/28	86 (92.5)	72/14
TPL	23 (24.2)	16/7	17 (18.3)	13/4
No TPL	72 (75.8)	40/32	76 (81.7)	65/11
PIH	9 (9.5)	5/4	6 (6.5)	5/1
No PIH	86 (90.5)	51/35	87 (93.5)	73/14

*CP, cerebral palsy; M, male; F, female; HIE, hypoxia ischemia encephalopathy; PVL, periventricular leukomalacia; MR, mental retardation; PROM, premature rupture of membrane; TPL, threatened premature labor; PIH, pregnancy-induced hypertension*.

The clinical data were stratified by the type of CP, birth asphyxia, and complications such as PVL and hypoxic–ischemic encephalopathy. The criteria for these risk factors were as previously described (28).

### Sample Collection

The blood samples were taken by skilled nurses. EDTA was routinely used as the anti-coagulant in the study. The samples were separated by centrifugation (1,500 × *g* for 15 min) at 21°C within 2 h after being collected. The plasma was obtained from the supernatant, and DNA was obtained from the remaining blood components in the same sample. All the fractions were stored at −80°C until use. All methods were performed in accordance with the relevant guidelines and regulations.

### Polymorphism Selection

Five SNPs (rs1554286, rs1518111, rs3024490, rs1800871, and rs1800896) of the *IL-10* gene whose minor allele frequencies in the Chinese Han population are more than 0.1 were selected from the dbSNP database^1^ and the HapMap human SNP database.^2^ The rs1554286 (intron 3), rs1518111 (intron 2), and rs3024490 (intron 1) SNPs are located in the coding region of *IL-10*, and the rs1800871 and rs1800896 SNPs are located in the upstream promoter region.

### Genotyping

After collecting the plasma, the AxyPrep Blood Genomic DNA Miniprep Kit (Axygen Biosciences, Union City, CA, USA) was used for preparing genomic DNA from the remaining blood components by following the manufacturer’s protocol. After the amplification of SNP-spanning fragments by multiplex PCR, the SNPs were genotyped on the Sequenom MassARRAY SNP genotyping platform (Sequenom, San Diego, CA, USA). The probes and primers were designed by the SEQUENOM online tools.^3^ The genotype results were analyzed by a person who was blinded to the clinical data.

### Cytokine Protein Level Measurement

The plasma samples for IL-10 protein analysis were thawed completely at room temperature, mixed by vortexing, and centrifuged at 1,500 × *g* for 15 min to collect the supernatants for the subsequent cytokine assay. A Milliplex Human Cytokine/Chemokine kit (IL-10, IL-17 IL-6, IL-8, TNF-α, and IFN-γ) was used with the Multiplex Cytokine Assay (Millipore, Billerica, MA, USA). Quality controls were performed in parallel between the plates using reagents provided in the kits. Data were acquired on a Luminex 200IS System (Luminex Corporation, Austin, TX, USA). There was a 6.94% plate variation in this assay, and the detection limit was 0.3 pg/mL. The samples with cytokine levels below the limit of detection were excluded. IL-10 levels were expressed as picograms per milliliter.

### Statistical Analysis

All gene analyses, including Hardy–Weinberg equilibrium tests, comparison of allele and genotype frequency, calculation of odd ratios and 95% confidence interval (95% CIs), estimation of pairwise linkage disequilibrium (LD), and haplotype association analysis were conducted with the SHEsis online software platform.^4^ The program SNPSpD,^5^ which takes marker LD information into consideration, was used to correct for multiple testing performed on each individual SNP. For IL-10 analysis, Student’s unpaired *t*-test was used. The Mann–Whitney *U*-test was used for the data with unequal variances. Statistical analyses were performed with SPSS (version 19.0) and Graphpad Prism 6.0 (Graphpad, La Jolla, CA, USA). All reported *p*-values were two-tailed, and statistical significance was set at *p* < 0.05.

## Results

### Association of *IL-10* Gene Polymorphisms With the Total Group With CP

To examine the association between CP and the polymorphisms in the *IL-10* gene, we detected genotype and allele frequencies of five SNPs in the 282 CP patients and the 197 healthy controls. The frequencies and analytic results for the SNPs are presented in Table 3. The genotypic distribution of the five selected SNPs of *IL-10* in the normal controls was in Hardy–Weinberg equilibrium. In the analysis of allele frequencies, differences between the total CP patients (*n* = 282) and normal controls (*n* = 197) for rs3024490 (*p* = 0.011, after SNPSpD correction, *p* = 0.033) and rs1800871 (*p* = 0.011, after SNPSpD correction, *p* = 0.033) were observed. There was also strong LD between rs3024490 and rs1800871 (*r*^2^ = 0.955) (Table 4), which makes it reasonable to have the same *p*-value for rs3024490 and rs1800871.

**Table 3:** Allele and genotype frequencies of *IL-10* in total cerebral palsy (CP) patients and controls.

Group	Allele frequency		*p* Value	*p΄* Value	OR (95% CI)	Genotype frequency			*p* Value	H–W
rs1554286	C	T				C/C	C/T	T/T		
CP	181 (0.322)	381 (0.678)	0.023		0.731 [0.558–0.958]	29 (0.103)	123 (0.438)	129 (0.459)	0.032	0.968
Control	152 (0.394)	234 (0.606)				36 (0.187)	80 (0.415)	77 (0.399)		0.067
rs1518111	A	G				A/A	A/G	G/G		
CP	388 (0.690)	174 (0.310)	0.018		1.387 [1.056–1.820]	132 (0.470)	124 (0.441)	25 (0.089)	0.017	0.589
Control	238 (0.617)	148 (0.383)				79 (0.409)	80 (0.415)	34 (0.176)		0.087
rs3024490	G	T				G/G	G/T	T/T		
CP	170 (0.306)	386 (0.694)	**0.011**	**0.033**	0.702 [0.533–0.924]	24 (0.086)	122 (0.439)	132 (0.475)	0.018	0.574
Control	145 (0.386)	231 (0.614)				32 (0.170)	81 (0.431)	75 (0.399)		0.214
rs1800871	C	T				C/C	C/T	T/T		
CP	162 (0.307)	366 (0.693)	**0.011**	**0.033**	0.696 [0.527–0.921]	25 (0.095)	112 (0.424)	127 (0.481)	0.018	0.966
Control	143 (0.389)	255 (0.611)				34 (0.185)	75 (0.408)	75 (0.408)		0.054
rs1800896	A	G				A/A	A/G	G/G		
CP	525 (0.934)	37 (0.066)	0.019		1.732 [1.091–2.751]	246 (0.875)	33 (0.117)	2 (0.007)	0.053	0.448
Control	344 (0.891)	42 (0.109)				153 (0.793)	38 (0.197)	2 (0.010)		0.832

*p′-Value, the p-value after the SNPSpD correction; The significance was set at p′<0.05 after correction; OR, odds ratio; CI, confidence interval*.

**Table 4:** The linkage disequilibrium among the SNPs in *IL-10*.

D′/*r*^2^	rs1554286	rs1518111	rs3024490	rs1800871	rs1800896
rs1554286		0.995	0.985	0.979	1.000
rs1518111	0.941		0.990	0.990	1.000
rs3024490	0.925	0.976		0.980	1.000
rs1800871	0.917	0.970	0.955		1.000
rs1800896	0.168	0.177	0.176	0.168	

*The standardized D′ values are shown above the diagonal, and the r^2^ values are shown below the diagonal*.

### Association of *IL-10* Gene Polymorphisms With the Subgroups of CP

Because CP is a complex syndrome with multiple etiological factors involved in disease pathogenesis, we performed subgroup analysis of *IL-10* SNPs according to subtypes of CP, birth complications, and maternal factors. The frequencies of allele and genotype were different between CP patients with spastic tetraplegia (*n* = 123) and normal controls (*n* = 197) for rs1554286, rs151811, rs3024490, rs1800871, and rs1800896 (*p_allele_* = 0.015, 0.009, 0.006, 0.003, and 0.006, respectively, after the SNPSpD correction), but no other significant differences were found when comparing allele and genotype distributions of these five SNPs between other CP subgroups and controls (Table 5).

**Table 5:** Allele and genotype frequencies of *IL-10* in cerebral palsy (CP) patient subgroups and controls.

Group	Allele frequency		*p*-Value	*p΄*-Value	OR [95% CI]	Genotype frequency			*p*-Value	*p΄*-Value	H–W
rs1554286	C	T				C/C	C/T	T/T			
CP + tetraplegia	70 (0.285)	176 (0.715)	**0.005**	**0.015**	0.612 [0.434–0.864]	9 (0.073)	52 (0.423)	62 (0.504)	**0.013**	**0.039**	0.671
Control	152 (0.394)	234 (0.606)				36 (0.187)	80 (0.415)	77 (0.399)			0.067
rs1518111	A	G				A/A	A/G	G/G			
CP + tetraplegia	180 (0.732)	66 (0.268)	**0.003**	**0.009**	1.696 [1.197–2.404]	64 (0.520)	52 (0.423)	7 (0.057)	**0.006**	**0.018**	0.395
Control	238 (0.617)	148 (0.383)				79 (0.409)	80 (0.415)	34 (0.176)			0.087
rs3024490	G	T				G/G	G/T	T/T			
CP + tetraplegia	64 (0.264)	178 (0.736)	**0.002**	**0.006**	0.573 [0.402–0.815]	7 (0.058)	50 (0.413)	64 (0.529)	**0.009**	**0.027**	0.494
Control	145 (0.386)	231 (0.614)				32 (0.170)	81 (0.431)	75 (0.399)			0.214
rs1800871	C	T				C/C	C/T	T/T			
CP + tetraplegia	60 (0.259)	172 (0.741)	**0.001**	**0.003**	0.549 [0.383–0.787]	7 (0.060)	46 (0.397)	63 (0.543)	**0.004**	**0.012**	0.713
Control	143 (0.389)	255 (0.611)				34 (0.185)	75 (0.408)	75 (0.408)			0.054
rs1800896	A	G				A/A	A/G	G/G			
CP + tetraplegia	236 (0.959)	10 (0.041)	**0.002**	**0.006**	2.881 [1.418–5.856]	113 (0.919)	10 (0.081)	0 (0.000)	**0.010**	**0.030**	0.638
Control	344 (0.891)	42 (0.109)				153 (0.793)	38 (0.197)	2 (0.010)			0.832

*p΄-Value, the p-value after the SNPSpD correction; The significance was set at p΄<0.05 after correction; OR, odds ratio; CI, confidence interval*.

### Cytokine Analysis

**Figure 1:**
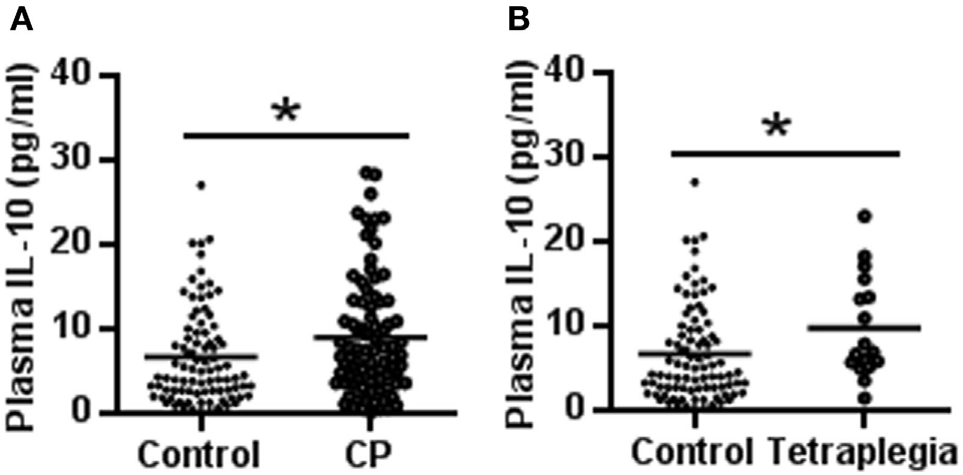
IL-10 concentration in cerebral palsy (CP) patients and controls. **(A)** The scatter plot of plasma IL-10 levels in CP patients and controls. **(B)** The scatter plot of plasma IL-10 in tetraplegia CP patients and controls (*p < 0.05).

The plasma IL-10 protein level was increased significantly in CP patients compared to normal controls (9.13 ± 7.07 vs. 6.79 ± 5.63 pg/ml, respectively, *p* = 0.017) (Figure 1A). The subgroup analysis showed that the plasma IL-10 level was also increased in the CP patients with spastic tetraplegia compared to the controls (9.86 ± 6.00 vs. 6.79 ± 5.63 pg/ml, respectively, *p* = 0.044) (Figure 1B). There were no significant differences in plasma IL-10 protein levels in other subgroup analyses.

### Association of the SNPs With Cytokine Production

**Figure 2:**
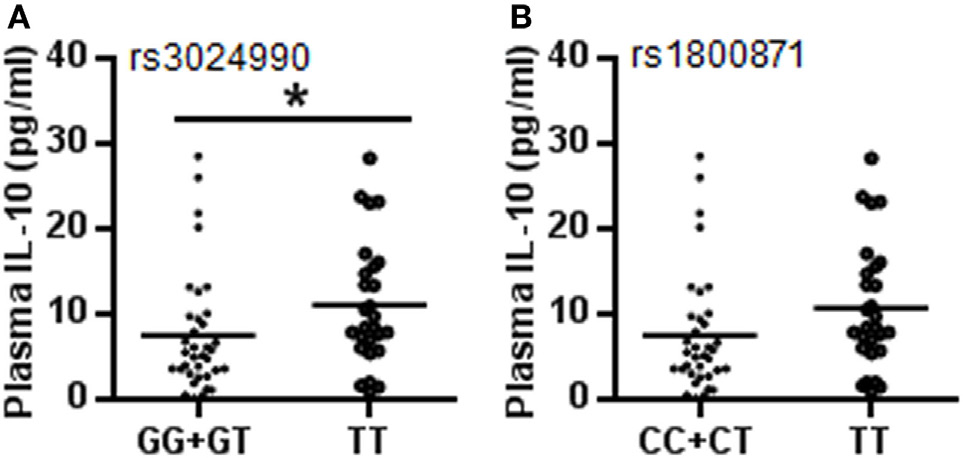
Plasma IL-10 levels with different genotypes at rs3024490 and rs1800871. **(A)** IL-10 levels with different genotypes at rs3024490. **(B)** IL-10 levels with different genotypes at rs1800871. Each dot represents one patient (*p < 0.05).

To confirm whether the IL-10 gene is linked with CP susceptibility, we further analyzed the relationship between genotypes of two SNPs associated with CP and plasma IL-10 protein levels. The results showed that the TT genotype had higher plasma IL-10 protein levels compared to the GG + GT genotype at rs3024490 (11.14 ± 7.27 vs. 7.44 ± 6.95 pg/ml, respectively, *p* = 0.045) (Figure 2A), and rs1800871 showed a tendency of high IL-10 protein level in the TT genotype compared to the CC + CT genotype (10.82 ± 7.33 vs. 7.62 ± 6.96 pg/ml, respectively, *p* = 0.079) in CP cases (Figure 2B), whereas no association was found for either SNP in controls. Although IL-10 is produced by constitutive or inducible expression, these results indicated that the genotypes of rs3024490 might play a more important role in the induced expression of IL-10.

## Discussion

It has been accepted that inflammation plays an important role in the brain during the perinatal period and might contribute to the development of CP (23). Several studies have reported that increased pro-inflammatory cytokines such as IL-1β, IL-6, TNFα, and IL-8 in cord blood or amniotic fluid are associated with CP (23, 29), and IL-6 protein levels are higher in the blood (28) and cerebrospinal fluid of CP patients (30). Moreover, the balance between the levels of pro-inflammatory and anti-inflammatory cytokines determines the effect of the inflammatory responses.

IL-10 function is complicated and can have both stimulatory and inhibitory effects on different types of immune responses. IL-10 can strongly inhibit the secretion of IL-2/IFN-γ by TH1 cells, and it inhibits the secretion of IL-1, IL-6, IL-12, and tumor necrosis factor in macrophages and dendritic cells in order to reduce tissue damage (31, 32). IL-10, as a potential anti-inflammatory cytokine, creates favorable conditions for the persistence of microbes and chronic diseases (33), both of which are involved in clinical perinatal brain damage (15). It has been reported that the upregulation of IL-10 after injury might have anti-inflammatory effects in specific regions of the immature brain in the postnatal rat brain infection model (34), and anatomical characteristics have been associated with functional findings in athetotic-type and spastic-type CP using an atlas-based analysis (35). In the current study, we found that IL-10 protein levels were increased in CP patients, especially in spastic tetraplegia, the most severe subtype of CP patients. The predominantly negative immune regulatory functions of IL-10 can play a critical role in mediating T-cell functional exhaustion, which makes these cells detrimental in chronic infection (36). Prolonged microglial reactivity and increased cytokine expression have been noted in animal models of traumatic brain injury, and altered inflammatory responses have been shown to persist for at least 7 years after brain damage in CP patients (37). This suggests that one reason for why the plasma IL-10 level increases in CP patients is the inability for the body to clear antigen, which leads to repetitive antigen stimulation and IL-10 induction in a manner similar to chronic infection. In other words, the high plasma IL-10 level might be an attempt by the immune response to counterbalance the expression of pro-inflammatory cytokines (38). Our present results support the hypothesis that inflammation triggers an inadequate immunological response in preterm infants with a consequently increased risk of CP. The cytokines, as indicators of inflammation, usually have a half-life of only a few hours, whereas previous studies (3, 7, 9, 10, 23, 29) and our study have shown persistent inflammation in CP patients. Therefore, it is possible that the inflammatory process in the brain is continuously being activated after perinatal brain injury, and future studies should focus on the dynamic changes in neuroinflammation and how these changes relate to the progression of CP.

Gene polymorphism studies suggest the involvement of as yet unidentified linkages between allelic variants and the pathogenesis of disease (39), and SNPs in genes encoding cytokines and their receptors have been implicated in both increased and decreased risk of perinatal brain injury (23). Studies have suggested that *IL-6, IL-8*, and *TNF-α* SNPs (40, 41) might predispose to CP because an increased risk for CP has been shown to be positively associated with the increased production of these proteins, but nothing has been known about the relationship between *IL-10* SNPs and CP.

The human *IL-10* gene is located on chromosome 1q31–1q32 and is composed of four introns and five exons (42). Three SNPs in the *IL-10* intron region (rs1554286, rs1518111, and rs3024490) have been reported to be associated with risk for ischemic stroke and idiopathic recurrent miscarriage (43–45), and two SNPs in the *IL-10* promoter region (rs1800871 and rs1800896) have been identified as highly polymorphic risk factors for systemic sclerosis (46) and Alzheimer’s disease (47). These five SNPs are in strong LD, and this is believed to have biological significance (39). In the current study, all five SNPs were associated with tetraplegia CP patients. In addition, reports have also shown that the upregulation of IL-10 after injury might have anti-inflammatory effects in distinct anatomic sites in the postnatal rat brain infection model (34), and anatomical characteristics have been delineated with functional findings in athetotic-type and spastic-type CP using the atlas-based analysis (35). In this study, we found the TT genotype frequency in the CP group to be higher than the control group and the TT genotype of rs3024490 and rs1800871 seems to be related to higher circulating levels of IL-10 protein in CP patients but not in controls. These results suggest that the genotype TT of rs3024490 associated an increased IL-10 expression might be due to the different reactivity of genetic variants to risk factors for CP (48, 49). Therefore, we hypothesize that cytokines affect different regions of the developing brain and that the subtypes of CP are determined by the functional genotypes of *IL-10* in the development of CP. Thus this group of children should be paid more attention to, especially in early life before clinical symptoms of CP begin to appear, which is exactly where the significance of the present study lies. However, we need to expand the sample size in subsequent experiments in order to test this hypothesis.

Cerebral palsy is a heterogeneous disease with complex interactions between genetic influences and environmental influences. Understanding the genes and gene–environment interactions in CP and the underlying cytokine-related mechanisms might lead to the development of new preventive and therapeutic strategies for CP. In summary, this study suggests that *IL-10* SNPs are strongly associated with CP through the regulation of *IL-10* gene function that leads to altered IL-10 production. Future work should focus on the interactions between genetic and cytokine-related mechanisms in the pathogenesis of CP. A better understanding of the potentially beneficial and detrimental roles of IL-10 in CP might not only help improve our understanding of CP pathogenesis, but might also help develop novel strategies for the prevention of CP.

## Ethics Statement

Approval for the study was obtained from the ethics committee of Zhengzhou University (201002006) in accordance with the Helsinki declaration.

## Author Contributions

CZ, QX, and XW conceived the project. LX, DB, MC, JS, XZ, YW, DZ, QS, and FX conducted the experiments. DB, LX, XW, QX, and CZ wrote the manuscript. All of the authors discussed the results and commented on the manuscript.

## Conflict of Interest Statement

The authors declare that the research was conducted in the absence of any commercial or financial relationships that could be construed as a potential conflict of interest.

## Footnotes

^1^www.ncbi.nlm.nih.gov/SNP (accessed December 18, 2014).

^2^www.hapmap.org (accessed March 10, 2015).

^3^http://agenabio.com/ (accessed June 5, 2015).

^4^http://analysis.bio-x.cn/ (accessed September 20, 2015).

^5^http://gump.qimr.edu.au/general/daleN/matSpD/ (accessed December 15, 2015)
